# Ulinastatin alleviates pulmonary edema by reducing pulmonary permeability and stimulating alveolar fluid clearance in a rat model of acute lung injury 

**DOI:** 10.22038/IJBMS.2022.64655.14230

**Published:** 2022-08

**Authors:** Yuan-xu Jiang, Ze-wei Huang

**Affiliations:** 1 Department of Anesthesiology, Shenzhen People’s Hospital (The Second Clinical Medical College, Jinan University, The Fist Affiliated Hospital, Southern University of Science and Technology), Shenzhen, Guangdong Province, 518020, P.R. China; 2 Department of Critical Care Medicine, Shenzhen People’s Hospital, Shenzhen, Guangdong Province, 518020, P.R. China

**Keywords:** Acute lung injury, Epithelial sodium channel, K-ATPases, Na, Pulmonary edema, Tight junction proteins, Ulinastatin

## Abstract

**Objective(s):**

Previous studies have shown that ulinastatin (UTI) alleviates pulmonary edema in acute lung injury (ALI) caused by lipopolysaccharide (LPS), although the mechanism behind this action is uncertain. This research aimed to identify the fundamental mechanism by which UTI alleviates pulmonary edema.

**Materials and Methods:**

We established a model of acute lung injury (ALI) in rats by using LPS as the inciting agent.The control, LPS, and LPS+UTI groups were each comprised of a specific number of randomly selected Wistar rats. We evaluated lung injury and determined pulmonary edema. The concentrations of TNF-α, IL-1β and IL-6 in BALF and the expression levels of α1Na, k-ATPase, β1Na, K-AtPase, α-ENaC, β-ENaC, γ-ENaC, Zonula occludens (ZO)-1, Occludin, Caludin-5, PI3K, Akt, TLR4, MyD88 and NF-ƘBwere identified in lung tissues.

**Results:**

The presence of UTI was associated with a reduction in lung pathological alterations, lung injury scores, the lung W/D ratio, and MPO activity, in addition to the improved gas exchange (P<0.01). Furthermore, UTI alleviated EB leakage and stimulated AFC (*P*<0.01). Importantly, UTI increased the expression of ZO-1, Occludin, Caludin-5, α1Na, K-ATPase, β1Na, K-AtPase, α-ENaC, β-ENaC, and γ-ENaC (*P*<0.01). Furthermore, UTI inhibited the inflammatory response, enhanced the expression of PI3K and Akt and hindered TLR4, MyD88, and NF-ƘB expression (*P*<0.01) in lung tissues.

**Conclusion:**

Our results demonstrated that UTI attenuated pulmonary edema by reducing pulmonary permeability and promoting AFC through inhibiting the inflammatory response, and the mechanism is related to promoting PI3K/Akt signaling pathways and suppressing TLR4/MyD88/NF-ƘB signaling pathways.

## Introduction

 Acute lung injury (ALI) marked by severe morbidity and mortality, is an initial manifestation of acute respiratory distress syndrome (ARDS) (1). The major treatments for ALI are lung-protective ventilation (2), ventilation in a prone position (3), corticosteroid use (4), neuromuscular blockage (5), and so forth. Despite enhanced treatment, the rate of death for ALI patients can reach 46% (6). No pharmaceutical medication now known for ALI could minimize early or late mortality. Thus, effective therapies for this possibly treatable lung injury must be discovered immediately.

Pulmonary edema is a hallmark of ALI (7). It is well established that pulmonary edema is essentially an imbalance in fluid production and clearance in the lung.Regular respiration depends on the exchange of oxygen and carbon dioxide through an alveolar-capillary barrier consisted of epithelial cells of alveolus and endothelium of pulmonary capillary. Tight junctions (TJs) contribute significantly to the formation of the alveolar-capillary barrier, and the disruption of TJs leads to increased permeability and pulmonary edema (8, 9). In addition, Na,K-adenosine triphosphatase (ATPase) and epithelial sodium channel (ENaC) proteins expressed in epithelium of alveoli are of great importance in promoting AFC (10, 11). Therefore, to reduce pulmonary edema in ALI, it is crucial to sustain TJ proteins, Na, K-ATPase, and ENaC expression.

Pulmonary edema could result from an inflammatory reaction. Multiple inflammatory cytokines, like TNF- and IL-1**β**, may damage the tight connections of capillary endothelial cells, as per recent studies. Hindering the production of inflammatory cytokines could also reduce lung permeability, as per studies (12, 13). In addition, TNF- and IL-1**β** may decrease alveolar epithelial cell functionality and lower Na,K-ATPase and ENaC expression (14). Suppression of the inflammatory reaction may therefore be advantageous for alleviating pulmonary edema by mitigating the damage to the pulmonary capillary barrier and enhancing AFC.

Urinary trypsin inhibitor ulinastatin, known as UTI, is a serine protease inhibition agent that is extracted from human urine. It was demonstrated to be useful in treating acute pancreatitis as well as acute circulatory failure in clinical testing. According to reports, the anti-inflammatory properties of UTI have beneficial effects on numerous organs (15-17)*. *Meanwhile, the method by which UTI diminishes pulmonary edema is still unknown. Therefore, to detect the effect that UTI has on pulmonary edema and investigate its potential pathway in LPS-induced ALI, this research employed LPS as an induction method for ALI.

## Materials and Methods

***Animals*** ***and grouping***

Guangdong Medical Animal Experiment Center supplied 18 Wistar rats of the SPF type, aged 6 to 8 weeks and weighing 180~220 g. (No. SCXK YUE 2019-0035). The rats were maintained in an animal care facility with a controlled environment, where food and water were available as desired and they were subjected to a constant cycle of dark and light 12 hr/12 hr. Sodium pentobarbital (50 mg/kg, IP) was injected into the rats’ left femoral veins through catheters (PE-50) for anesthetization. If needed, further doses of pentobarbital (about 1-3 mg/kg, IV) were administered to prolong anesthesia. 

The rats were categorized by random into 3 groups (n=18): control, LPS, and LPS+UTI. The control group rats were given 5 ml/kg of normal saline. To induce ALI, LPS (10 mg/kg) was administered into the femoral vein (Sigma Chemical Company, St. Louis, MO, USA). Thirty min after receiving LPS, the LPS+UTI groupings rats were infused with 20,000 U/kg IP dosage of UTI. Consistent with earlier researches (18) and our initial investigations, the UTI dose was calculated (data not present). 24 hr following LPS injection, the rats were put to death by a method known as bloodletting. Then samples of their blood, bronchoalveolar lavage fluid (BALF), and lung samples were gathered for further examination. The testing methods were authorized by the Animal Care Committee of Shenzhen People’s Hospital and carried out in compliance with institutional rules for animal care and the National Institutes of Health’s Guide for the Care and Use of Laboratory Animals.

***Histological examinations***

 Lung samples were retrieved 24 hr following LPS injection. The right lung inferior lobe was retrieved, and the specimens were paraffin-embedded and regularly preserved. Hematoxylin and eosin (H&E) stained samples for light microscopy investigation. Through the use of light microscopy, the intensity of the histological lung injury in five random fields was determined on the basis of congested alveoli, bleeding, neutrophil infiltration rate into the airway or capillary wall, alveolar wall thickness, and alveolar barrier thickness were graded blindly. Lung samples were evaluated as 1 (no or minor pathological alterations), 2 (little pathological alterations), 3 (average pathological alterations), or 4 (extensive pathological alterations). The rating scores were counted making up the total injury score.

***Measurements of the lung wet/dry weight ratio (W/D)***

After the rats were executed, an instantaneous removal of thorax was conducted, the right lung was excised to retrieve the upper lobe, following by weighting the moist weight of the represented animals. After that, it was set in a kiln preheated to 75 °C and dried in it for 24 hr; the determined weight indicates the dry mass. The degree of pulmonary edema was evaluated by measuring the ratio of lung W/D.

***Measurement of arterial oxygen partial pressure (PaO***_2_ ***), arterial carbon dioxide partial pressure (PaCO***_2_ ***), oxygenation index (PaO***_2_ ***/FiO***_2_ ***)***

 Each rat’s right common carotid artery was used to withdraw a blood sample (1 ml) 24 hr after LPS administration, and PaO_2_, PaCO_2_, and PaO_2_/FiO_2_ were instantly analyzed by utilizing a blood gas analyzer (Stat Profile pHOx, Nova Biomedical Corporation; Waltham, MA, USA).

***Myeloperoxidase (MPO) activity analysis***

The lung samples were mashed and centrifuged, after tha an incubation of the supernatant in a water bath (60 °C) for 2 hr was conducted to assess MPO activity as per the producer ‘s guidelines of the assay kits (Nanjing Jianchen Bioengineering Institute, Nanjing, China).

***Measurement of pulmonary capillary leakage***

A total of 18 rats were subjected to pulmonary capillary permeability. In a brief, at 24 hr after the administration of LPS, an Evans blue dye solution having 2.5% concentration and 20 mg/kg diluted in 1 ml of normal saline was infused into the femoral vein. Using a cannula inserted into the thoracic trunk, surplus Evans blue was eliminated from the pulmonary arteries 30 min after the injection of Evans blue. This was accomplished by perfusing the lungs with phosphate buffered saline (PBS) (50 ml) over five min. The middle lobes of the right lung were excised and dehydrated for 72 hr at 60 °C. For Evans blue dye extraction, tissues were dissected, then formamide (4 ml/g) was added, and incubated at room temperature for 24 hr before being homogenized at 4000×g for 30 min. A microplate reader (wavelength, 620 nm) measured the stain absorbance score in the solution. A standard curve calculated how much Evans Blue dye was in 100 mg of lung sample. At least three separate instances of each experiment were carried out.

***Measurement of alveolar fluid clearance (AFC)***

AFC was calculated by monitoring the concentration of Evans blue-labeled albumin in 18 rats. To start, 5 ml/kg of an Evans blue-labeled bovine serum albumin perfusion solvent (Sigma Chemical Company, St. Louis, MO, USA) was infused into the left lung through the trachea, followed by 2 ml of oxygen to assist diffusion. Throughout the standard period, the rats were supplied with 100% oxygen, and we sustain lung pressure by maintaining their positive end expiratory pressure at 2 to 3 cm H_2_O. All cellular units were covered in plastic wrap before being incubated for 1 h in a 37 °C water bath. As soon as the alveolar fluid was aspirated, a spectrophotometer was used to measure the marked albumin at 620 nm. AFC was calculated using the following equation: AFC (%) = [(Cf -Ci)/Cf]100%, where Ci stands for Evans blue-marked % albumin concentration that was injected and Cf stands for the ultimate concentration of 5% albumin.

***Detection of cytokine concentrations in BALF***

Following the execution of the rats, the major bronchus was dissected. In addition to ligating the right bronchus, a customized tracheal catheter was introduced through the major bronchus. After that, 2 ml of cold PBS were injected into the left lung, and it was flushed three times. The bronchoalveolar lavage fluid (BALF) was centrifuged for 10 min at a g-force at 1200×g a temperature of four degrees Celsius. The supernatant was portioned out into aliquots before being frozen at a temperature of -70 ° C. ELISA was employed to detect the amounts of TNF-α, IL-1**β**, and IL-6 in BALF supernatant in compliance with the producer’s recommendations (Shanghai Jianglai Biotechnology Co., Ltd, Shanghai, China). Serum values of TNF-α, IL-1**β**, and IL-6 were determined by ELISA kits in accordance with the supplier’s guidelines, using blood samples from the main arteries (Shanghai Jianglai Biotechnology Co., Ltd, Shanghai, China).

***Western blot analysis***

With RIPA lysis buffer (50 mM Tris [pH 7.4], 150 mM NaCl, 1% Triton X-100, 1% sodium deoxycholate, 0.1 % SDS, sodium orthovanadate, sodium fluoride, EDTA, and leupeptin) and PMSF, proteins were retrieved. Utilizing a BCA protein assay kit, the protein levels in the supernatants were measured. Using sodium dodecyl sulfate–polyacrylamide gel electrophoresis (SDS–PAGE), the products were extracted and deposited to PVDF membranes. The membrane was blocked for 1 h at room temperature with a tris buffer solvent having 5% skim milk powder. The membrane was therefore filtered with PBS having 0.5% Tween 20 and rinsed five times with PBST (5 min every time). Therefore, the membrane was incubated with the primary antibodies listed below at a temperature of 4 ° C for a period of one night: anti-α1Na,KATPase antibody (CST, Boston, MA, USA), anti-β_1_Na,KATPase antibody (CST), anti-α-ENaC (CST), anti-β-ENaC (CST), anti-γ-ENaCP (CST), anti-ZO-1antibody (CST), anti-Occludin antibody (CST), anti-Claudin antibody (CST), anti- PI3K (CST), anti-Akt (CST), anti-TLR4 (CST), anti- MyD88 (CST), anti-NF-ƘB antibody (CST) and anti-β-actin antibody (Bioworld Technology, Minneapolis, MN, USA). After a three-hour of incubation at room temperature with a horseradish peroxidase (HRP)-labeled goat anti-rabbit antibody (Abcam, Cambridge, UK), the membrane was rinsed five times with PBST to remove any remaining antibody (5 min every time). After that, the bands were subsequently seen by utilizing a UVP gel imaging equipment in conjunction with an enhanced chemiluminescence kit (ECL) (Upland, CA, USA). ImageJ software was employed to examine the band intensities (National Institutes of Health, USA).

***Statistical analysis***

SPSS 19.0 analyzed all the data. According to a normal distribution, measurement information is expressed as the mean ± standard deviation (mean ± SD). One-way ANOVA was utilized to examine the variations among the groups, whereas Dunnett’s test was utilized to contrast the two groups. *P*<0.05 was judged as statistically significant.

## Results

***Ulinastatin mitigated LPS-induced ALI in rats***

**Figure 1:**
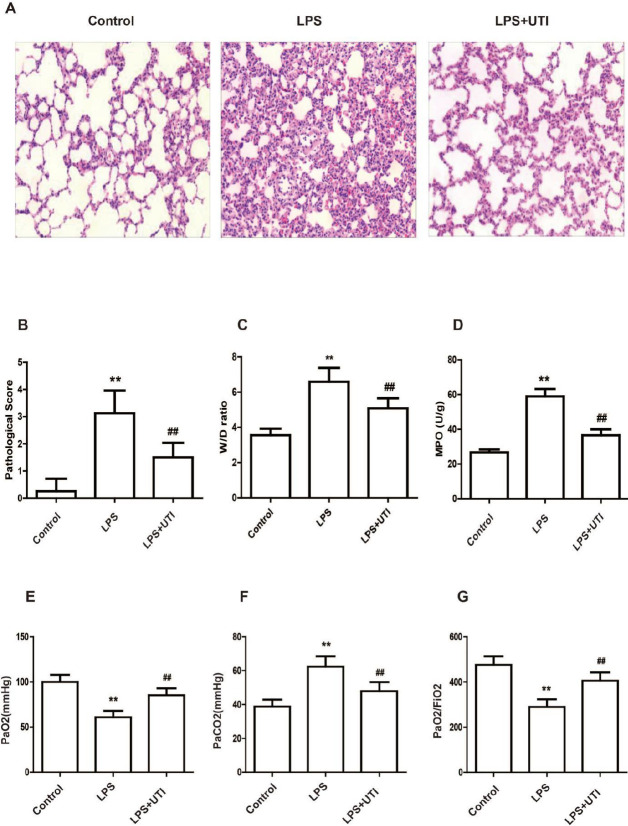
Ulinastatin mitigated LPS-induced acute lung injury

First, HE staining was utilized to visualize pathological lung alterations generated by LPS. In the non-experimental or control group, the pulmonary structures were unharmed and lacked of inflammatory cells infiltrations into the alveolar cavity. The increase in the lung damage score demonstrates this; LPS generated significant alterations in lung injury, such as interstitial edema in comparison with the control group, alveolar barrier thickness, and a huge volume of inflammatory cell infiltration. The LPS-induced morphological changes and lung injury scores were significantly reduced by UTI treatment (Figure 1A-B, *P*<0.01). Additionally, we investigated the lung W/D ratio in addition to the MPO activity in the lung samples, and the results showed that UTI therapy significantly diminished the lung W/D ratio in addition to the MPO activity relative to the LPS group (Figure. 1C-D, P 0.01). We also performed a blood gas analysis, which showed that UTI treatment enhanced PaO_2_ and PaO_2_/FiO_2 _and reduced PaCO_2 _(Figure 1E-G, *P*<0.01).

***Ulinastatin reduces alveolar capillary permeability***

**Figure 2:**
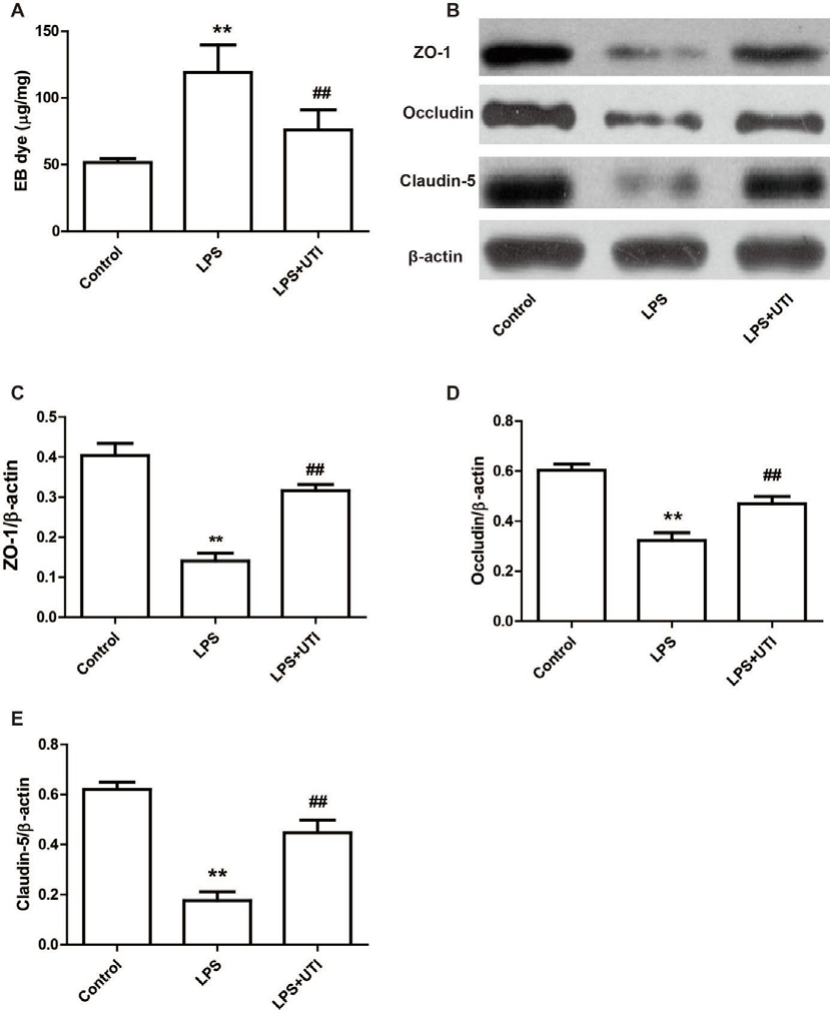
Ulinastatin reduces lung permeability by increasing the expression of ZO-1, Occludin and Claudin-5 in LPS-induced acute lung injury

Alveolar capillary barrier damage was evaluated by calculating the EB concentration in BALF. Of note, UTI significantly decreased the EB concentration in the LPS+UTI group comparing with the LPS group (Figure 2A, *P*<0.01).

***Ulinastatin*** **enhanced the expression of** ***ZO-1, Occludin, and Caludin-5 in LPS-induced ALI in rats***

We evaluated the expression of ZO-1, Occludin, and Calludin-5 in lung samples to further clarify the processes by which UTI decreases lung permeability. The expression of ZO-1, Occludin, and Claudin-5 was lower in the LPS group compared with the control group, however it was significantly higher in the LPS+UTI group relative to the LPS group (Figure 2B-E, *P*<0.01). These findings imply that UTI could minimize pulmonary edema by decreasing lung permeability.

***Ulinastatin promotes alveolar fluid clearance (AFC)***

**Figure 3:**
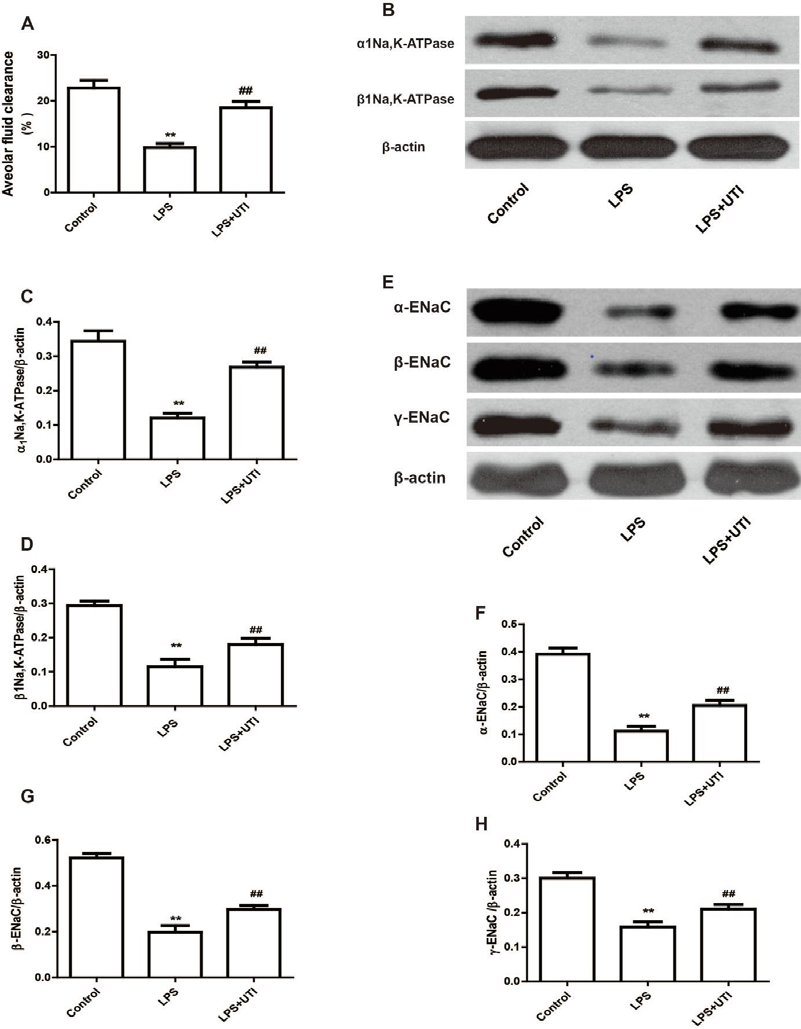
Ulinastatin promotes alveolar fluid clearance by increasing the expression of Na, k-ATPase and ENaC in LPS-induced acute lung injury

 Additionally, we studied the impact of UTI on AFC. AFC was reduced in the LPS group comparing with the control group; however, it was significantly elevated in the LPS+UTI group comparing with the LPS group (Figure 3A, *P*<0.01).

***Ulinastatin*** **enhanced the expression of** ***α***_1_ ***Na, k-ATPase, β***_1_ ***Na, K-AtPase, α-ENaC, β-ENaC, and*** ***γ*** ***-ENaC in LPS-induced ALI in rats***

Western blotting was employed to evaluate the expression of 1Na, k-ATPase, 1Na, K-AtPase, -ENAC, -ENaC, and -ENaC in lung tissues to determine whether UTI relieves pulmonary edema by boosting AFC. The expression of 1Na, k-ATPase, 1Na, K-AtPase, -ENAC, -ENAC, and -ENAC was diminished in the LPS groupings relative to the control groupings (Figure. 3B-H, P 0.01), while significantly elevated in the LPS+UTI groupings comparing with the LPS groupings (Figure. 3B-H, P <0.01). These findings imply that UTI could decrease pulmonary edema by boosting AFC.

***Ulinastatin attenuated the inflammatory response in LPS-induced ALI in rats***

**Figure 4:**
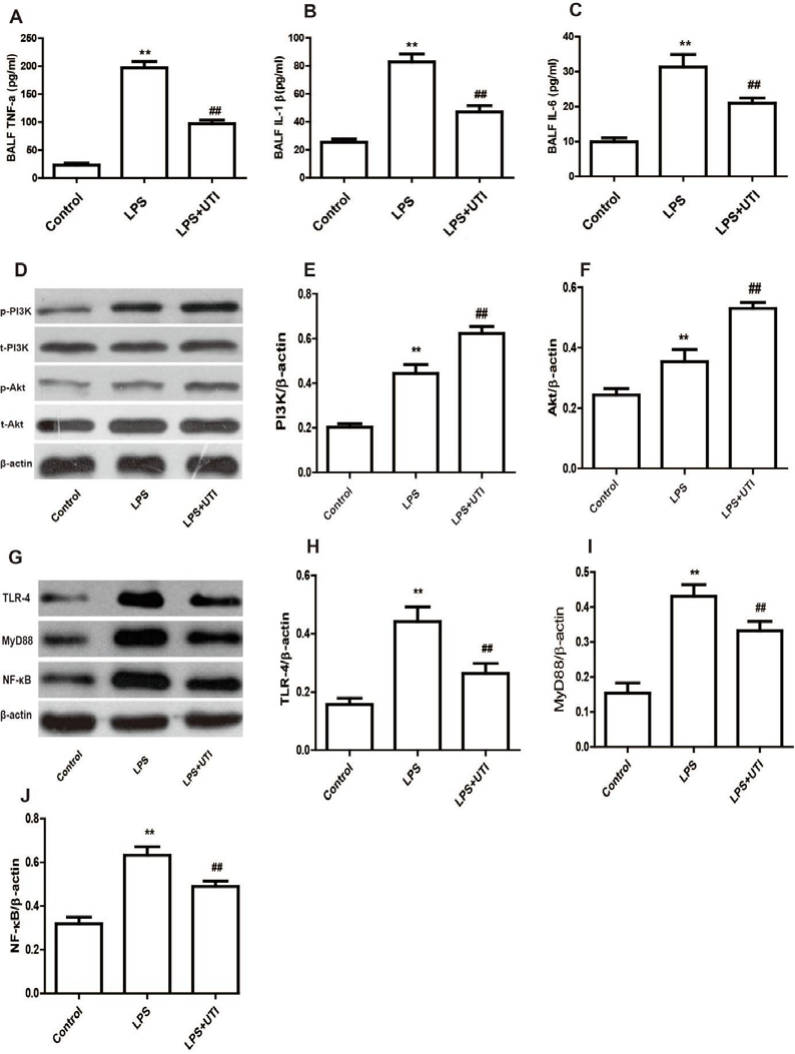
Ulinastatin attenuates the inflammatory response by stimulating the PI3K/Akt signaling pathway and inhibiting the TLR4/MyD88/NF-ƘB signaling pathway in LPS-induced acute lung injury

It is well known that the inflammatory response not only increases alveolar capillary permeability but also damages alveolar epithelial cells and impairs their ability to clear alveolar fluid. Therefore, we measured the effect of UTI on the concentrations of TNF-α, IL-1β and IL-6 in BALF and in BALF. The values of TNF-α, IL-β, and IL-6 were significantly higher in the LPS group comparing with the control group (Figure 4A-C, *P*<0.01), but significantly lower in the LPS+UTI group comparing the LPS group.

***Ulinastatin promotes the PI3K/Akt signaling pathway in LPS-induced ALI in rats***

To comprehend how UTI inhibits the production of inflammatory cytokines, we examined the PI3K/Akt signaling pathway. p-PI3K and p-Akt expression was found to be higher in the LPS groupings than in the control groupings, even though it was significantly higher in the LPS+UTI groupings than in the LPS groupings (Figure 4D-F, *P*<0.01).

***Ulinastatin inhibits the TLR4/MyD88/NF-*** *Ƙ* ***B signaling pathway in LPS- induced ALI in rats***

The impact of UTI on the expression of TLR4, MyD88, and NF-B was then investigated. The expression of TLR4, MyD88, and NF-ƘB was raised in the LPS groupings relative to the control groupings, but decreased significantly in the LPS+UTI group relative to the LPS groupings. Figure 4H-J, *P*<0.01, demonstrates that Dex reduced the inflammatory reaction caused by LPS by increasing PI3K/Akt and reducing the TLR4/MyD88/NF-ƘB signaling pathway.

(A) Representative microscopic findings of the lung tissues (HE staining). (B) Lung injury scores, (C) lung W/D ratio, (D) MPO activity, (E) PaO_2_, (F)PaCO_2_, (G) PaO_2_/FiO_2_. Data are expressed as the mean±SD (n=8). **P<0.01 vs. the control group; ##*P*<0.01 vs. the LPS group

LPS: Lipopolysaccharide; MPO: Myeloperoxidase

(A) Concentration of EB in BALF. (B-E) The expression levels of ZO-1, Occludin and Claudin-5 were assessed by Western blotting. Data are expressed as the mean±SD (n=8). **P<0.01 vs. the control group; ##*P*<0.01 vs. the LPS group

ZO: Zonula occludens; LPS: Lipopolysaccharide

(A) Alveolar fluid clearance. (B-D) The expression levels of α1Na, K-ATPase and β1Na, K-ATPase were assessed by Western blotting. (E-H) The expression levels of α-ENaC, β-ENaC, and γ-ENaC were assessed by Western blotting. Data are expressed as the mean±SD (n=8). **P<0.01 vs. the control group; ##*P*<0.01 vs. the LPS group

LPS: Lipopolysaccharide

(A-C) The concentrations of TNF-α, IL-1β, and IL-6 in BALF. (D-F) The expression of PI3K and Akt was assessed by Western blotting. (G-J). The expression of TLR4, MyD88, and NF-ƘB was assessed by Western blotting. Data are expressed as the mean±SD (n=8). ** *P*<0.01 vs. the control group; ##*P*<0.01 vs. the LPS group

TLR: Toll-like receptor; LPS: Lipopolysaccharide; BALF: Bronchoalveolar lavage fluid

## Discussion

Our research declared that UTI diminished pulmonary edema in rats with LPS-provoked ALI. Remarkably, UTI suppressed the Evans blue (EB) levels in BALF and promoted AFC. In addition, UTI mitigated the disruption of tight junction proteins (TJs) and elevated the Na, k-ATPase and ENaC expression. Furthermore, our research also revealed that UTI alleviated the inflammatory response induced by LPS by promoting PI3K/Akt and limiting the TLR4/MyD88/NF-ƘB signaling pathway. The above findings proved that UTI reduced pulmonary edema by decreasing lung permeability and increasing AFC by boosting TJ proteins and Na, k-ATPase and ENaC expression, respectively. Its mechanisms may be related to inhibiting the inflammatory response by promoting PI3K/Akt and reducing the TLR4/MyD88/NF-ƘB signaling pathway.

In normal lungs, fluid and small proteins cannot enter the alveolar cavity because of the alveolar capillary barrier (ACB), which facilitates the oxygen (O_2_) and carbon dioxide (CO_2_) exchanging. The stability of the barrier is ensured by proper TJs between its components. TJs comprise a variety of transmembrane proteins, the most well recognized of which are zonula occludens-1 (ZO-1), Occludin, and Calludin-5, which are dispersed predominantly in epithelial cells of alveoli and endothelial cells of pulmonary capillaries. Accumulated evidence suggests that reduced TJ protein expression leads to increased lung leakage (8, 12, 19). It is worth noting that UTI reduces pulmonary capillary permeability by protecting TJ proteins such as ZO-1, Occludin and Claudin-5.(13, 20). In other animal studies, traumatic brain injury (TBI) causes a reduction in the expression of ZO-1 as well as claudin-5, while UTI treatment reduces the interference with the blood brain barrier by elevating the expression of these proteins (21). The present results indicated Relative to the control group, the expression of TJ proteins like ZO-1, Occludin, and Claudin-5 was reduced in ALI produced by LPS. Our investigation demonstrated that UTI elevated the expression of ZO-1, Occludin, and Claudin-5, which is a positive result. These results imply that the breakdown of the pulmonary capillary barrier is a significant cause of pulmonary edema. and that UTI may be effective in alleviating pulmonary leakage by upregulating TJ protein expression.

Alveolar epithelial cells not only contribute in the formation of the pulmonary capillary barrier but are also essential in promoting alveolar AFC, thus providing optimal gas exchange (22). However, AFC is impaired in addition to the disruption of the pulmonary capillary barrier in patients with ALI, resulting in severe hypoxemia and worse outcomes (7). AFC is involved in alveolar epithelial cell Na+ transport. Na+ is primarily taken up through the ENaC at the summit epithelial cells of alveoli and is subsequently released into the lung interstitium by Na, K-ATPase and basolaterally positioned Na/K-ATPase, so establishing a local osmotic gradient to reuptake the edematous fluid from the alveolar cavities (23). Na,K-ATPase and ENaC play key functions in promoting AFC, and overexpression of Na,K-ATPase and ENaC can increase AFC and decrease pulmonary edema, according to previous research (10, 11, 24). According to these results, LPS suppressed the expression of Na,K-ATPase and ENaC and decreased AFC. Prior research has found that UTI mitigates pulmonary edema by decreasing lung leakage (13, 25). It is uncertain, if UTI relieves pulmonary edema via increasing AFC. According to our findings, UTI therapy enhances Na,K-ATPase and ENaC expression and activates AFC. The above results indicated that UTI reduces pulmonary edema by stimulating AFC as well as reducing lung permeability in LPS-induced ALI.

It is widely acknowledged that inflammation serves a crucial function in ALI (26). Prior research demonstrated that inhibition of the inflammatory response can reduce intestinal barrier and brain barrier dysfunction and reduce edema by upregulating TJ protein expression (27, 28). In addition, TNF-α alters the structure and function of lung TJs, leading to lung leakage, whereas TNF-α inhibitors alleviate lung leakage by upregulating the expression of ZO-1, Occludin and Claudin-5 (13, 29). TNF-α and IL-1β limit the expression of Na,K-ATPase and ENaC and decrease AFC (30, 31). These findings suggested that limiting the release of inflammatory cytokines may be advantageous for preserving the stability of the pulmonary capillary barrier and increasing AFC, hence preserving lung fluid balance. Nonetheless, it will be intriguing to determine if UTI elevates the expression of ZO-1, occludin, claudin-5, Na,K-ATPase, and ENaC by reducing the production of cytokines. In a number of clinical conditions, like infections, hemorrhagic shock, and ischemic–reperfusion, it was observed that UTI can inhibit the inflammatory reaction of lung tissue (32, 33). LPS raised the values of TNF-α, IL-6, and IL-1β in BALF and the activity of MPO in lung tissues, but UTI therapy reduced significantly these levels and activities, showing that UTI decreases the LPS-related inflammatory reaction. By reducing the inflammatory reaction, we hypothesize that UTI increased the expression of ZO-1, Occludin, and Claudin-5, Na, K-ATPase, and ENaC.

To further explore the molecular mechanism, we studied the PI3K/Akt and NF-KB signaling pathways. The lipid kinase PI3K creates the second messenger phosphatidylino- sitol (3, 4, 5)-trisphosphate (PIP3), which enhances the translocation of Akt to the cell membrane. Akt is phosphorylated at the membrane and has an essential impact in activities including cell growth, development, survival, and death. Notably, active PI3K/Akt inhibits the generation of inflammatory cytokines in vivo and in vitro, hence exerting anti-inflammatory actions (34, 35). In addition, UTI reduces inflammation generated by LPS in murine macrophage RAW264.7 cells through stimulating the PI3K/Akt/Nrf pathway (36). Our study showed that UTI upregulates PI3K and Akt expression, indicating that the PI3K/Akt signaling pathway could be important in modulating the inflammatory response.

 Toll-like receptor-4 (TLR4), which is found on the surface of monocytes and macrophages, is the primary receptor for endotoxin (LPS) and is essential for immune system stimulation (37). After LPS binds to TLR4, MyD88 is activated, facilitating the translocation of NF-ƘB from the cytoplasm to the nucleus and triggering the transcription of inflammatory genes. Growing research indicates that stimulation of the TLR4/MyD88/NF-B signaling pathway regulates the inflammatory reactions and promotes ALI (38). Other investigations demonstrated that UTI decreases the inflammatory response generated by LPS ALI via TLR4/NF-ƘB. (32). Our investigation revealed that LPS enhances the expression of TLR4, MyD88, and NF-ƘB, whereas UTI inhibits LPS-generated TLR4/MyD88/NF-B initiation. Accordingly, by hindering the TLR4/MyD88/NF-B signaling pathway, UTI may mitigate the inflammatory reaction in LPS-induced ALI. 

## Conclusion

Our research reveals that UTI decreases pulmonary edema by decreasing pulmonary permeability and increasing alveolar fluid clearance by overexpressing TJ proteins,Na,K-ATPase, and ENaC. In ALI generated by LPS, these preventive benefits occur in part by decreasing the inflammatory reaction through increasing PI3K/Akt and suppressing the TLR4/MyD88/NF-B signaling pathway. Our findings shed fresh light on the treatment of pulmonary edema and indicate a novel possible treatment for UTI in patients with ALI.

## Authors’ Contributions

YJ Wrote this article, YJ Worked on the experimental design. YJ and ZH Conducted the experiments, analyzed the data. All authors read and approved the final manuscript.

## Conflicts of Interest

The authors declare that they have no competing interests.
